# Progressive abnormal gait in an adult Jack Russell Terrier with a homozygous frameshift variant in *SETX* (senataxin)

**DOI:** 10.1093/jvimsj/aalag085

**Published:** 2026-05-09

**Authors:** G Diane Shelton, Sofie F M Bhatti, Luc Van Ham, Steven G Friedenberg, Jonah N Cullen, Ling T Guo, Katie M Minor

**Affiliations:** Department of Pathology, University of California San Diego, La Jolla, CA, United States; Faculty of Veterinary Medicine, Small Animal Department, Ghent University, Salisburylaan 133, Merelbeke 9820, Belgium; Faculty of Veterinary Medicine, Small Animal Department, Ghent University, Salisburylaan 133, Merelbeke 9820, Belgium; Department of Veterinary Clinical Sciences, College of Veterinary Medicine, University of Minnesota, St. Paul, MN 55108, United States; Department of Veterinary Clinical Sciences, College of Veterinary Medicine, University of Minnesota, St. Paul, MN 55108, United States; Department of Pathology, University of California San Diego, La Jolla, CA, United States; Department of Veterinary Clinical Sciences, College of Veterinary Medicine, University of Minnesota, St. Paul, MN 55108, United States

**Keywords:** genetic disease, movement disorder, muscle, myopathy, neuromuscular disease, whole genome sequencing

## Abstract

Here we describe an 8-year-old intact male Jack Russell Terrier with a 9-month history of slowly progressive gait disturbances that advanced over 2 years to generalized stiffness without ataxia and severe bilateral hyperflexion of all limbs. Gait evaluation showed high lifting of both pelvic limbs resulting in a hopping movement in the rear end; the right pelvic limb was kept lifted, making the dog trot on the 3 other limbs. Both thoracic limbs were lifted higher than normal. Complete blood count and serum biochemistry analysis including creatine kinase activity were normal. Electrophysiological examination showed no abnormalities. Muscle biopsy samples collected at a later stage of the disease showed no abnormalities. A homozygous frameshift variant in *SETX* (senataxin) was identified and is predicted to truncate about 85% of the protein. The *SETX* variant adds a new gene variant to the list affecting Jack Russell Terriers with gait abnormalities.

## Introduction

Movement disorders are a heterogeneous group of clinical neurological syndromes characterized by involuntary movements without loss of consciousness and can include myokymia (undulating muscle contractions), neuromyotonia (muscle stiffness), and paroxysmal dyskinesia among other abnormal movements.^1^ These various syndromes can be hard to classify clinically, and final classification might depend on gene variant identification.

In one of the earliest papers, myokymia and neuromyotonia were described in a group of 37 young Jack Russell Terriers (JRTs) beginning at 8 months of age.^2^ Most of these dogs exhibited clinical signs of spinocerebellar ataxia. In 2014, a homozygous missense variant in *KCNJ10*, the inwardly rectifying potassium channel, was described in young JRTs with early-onset spinocerebellar ataxia and myokymia.^3^ At present, it is known that the *KCNJ10* variant in the JRT is strongly associated with spinocerebellar ataxia.^4^ Not all JRTs show myokymia or neuromyotonia. In addition, dogs presenting only with myokymia or neuromyotonia (without spinocerebellar ataxia) did not carry the reported *KCNJ10* variant.^4^

### History, clinical signs, and diagnostic evaluations

An 8-year-old intact male JRT weighing 12 kg presented to the Faculty of Veterinary Medicine, Small Animal Department, Ghent University with a 9-month history of slowly progressive gait disturbances. The owners reported that before the onset of these signs, no abnormalities had been detected in the dog. They had no information regarding the littermates, dam, or sire. The dog was on an appropriate commercial diet, up to date on vaccinations and deworming, and had no travel history. Treatment by the referring veterinarian included administration of vitamin B-complex and carprofen with no apparent improvement.

On presentation, physical examination showed a well-muscled dog with no other abnormalities. On neurological examination, the dog’s gait at a trot showed high lifting of both pelvic limbs, creating a hopping movement with the rear end; the right pelvic limb was kept lifted at some points, making the dog trot on the 3 other limbs; both thoracic limbs were also lifted higher than normal (Video S1). When walking slowly, the dog showed hyperflexion of all 4 limbs. Weakness, ataxia, or exercise intolerance were not apparent. The remainder of the neurological examination (spinal reflexes, postural reactions, mentation, and cranial nerves) was normal. Spinal pain could not be elicited. Neurolocalization was difficult; however, a muscular problem was suspected. Complete blood count was normal, and serum biochemistry showed mild changes in glucose (5.22 mmol/L; 3.05-4.99) and potassium (3.8 mmol/L; 4-5.5) concentrations. Creatine kinase (CK) activity was normal (110 IU/L; < 321), as were liver function tests, sodium, calcium, magnesium, urea, creatinine, and total protein concentrations. Electrophysiological examination showed no abnormalities (including electromyography [EMG], motor nerve conduction velocity, compound muscle action potential amplitudes, sensory nerve conduction velocity, and repetitive nerve stimulation). Collection of muscle biopsy samples was advised but the owners refused further diagnostics at this stage.

One year after the initial presentation, the dog was presented for reassessment and additional diagnostics. The gait abnormalities had progressively worsened. The dog walked in slow-motion with severe hyperflexion of all 4 limbs (Video S2a). At a trot, both pelvic limbs were severely hyperflexed, leading to bunny-hopping, and the right pelvic limb was regularly lifted as if in a cramp (Video S2b). The remainder of the physical and neurological examinations was relatively unchanged; however, the dog showed mild muscle atrophy, mainly in both pelvic limbs, and had weak patellar reflexes bilaterally with decreased hopping reactions in the right pelvic limb. The same blood variables were measured, revealing normal CK activity (297 IU/L; < 321) and an abnormal hematocrit (39.7%; 43-57). Total T4, TSH, cholesterol, and triglyceride concentrations were normal. Muscle biopsy specimens were collected under general anesthesia (m. triceps brachii under refrigeration and in formaldehyde) and sent to the Comparative Neuromuscular Laboratory, University of California San Diego, by an express service. Upon receipt, the unfixed chilled biopsy was snap-frozen in isopentane precooled in liquid nitrogen, and cryosections were evaluated by a standard panel of histochemical stains and reactions.^5^ No specific abnormalities were identified in the biopsies from the triceps brachii muscle.

The dog was lost to further follow-up; however, a video update by the referring veterinarian taken 2 years after the initial presentation was submitted, showing clear neurological deterioration (Video S3). The dog showed severe hyperflexion of both pelvic limbs, leading to a bumping-bouncing gait and cramps in the right pelvic limb, making it difficult for the dog to walk. In addition, both thoracic limbs were hyperflexed.

### Genetic testing

Genomic DNA was prepared from archived frozen triceps brachii muscle using the Qiagen DNEasy kit according to package instructions. DNA libraries were prepared using an Illumina TruSeq PCR-Free kit, and 150 base pair (bp) paired-end reads were generated on an Illumina NovaSeq 6000 sequencer by Azenta Life Sciences (South Plainfield, NJ 07080). A total of 615 million paired-end reads were generated, corresponding to a mean 37-fold genome-wide coverage. Sequence reads were mapped against the dog reference genome UU_Cfam_GSD_1.0,^6^^,^^7^ concatenated with the Y chromosome from ROS_Cfam_1.0 (NCBI RefSeq GCF_014441545.1, available on NCBI’s short-read archive at PRJNA615959), and processed using the OnlyWAG pipeline as described.^8^ Raw sequence reads are available under NCBI BioProject PRJNA937381 (SRR34167788) (https://dataview.ncbi.nlm.nih.gov/object/PRJNA937381).

Whole genome sequence (WGS) variants from the affected dog were compared to an internal WGS database developed at the University of Minnesota Canine Genetics Laboratory containing 3023 dogs, wolves and coyotes of 402 diverse breeds; this database includes 1971 dogs, wolves, and coyotes released by the Dog10K consortium.^7^ The dataset included 5 Parson Russell Terriers but no other JRTs or Russell Terriers. The WGS data from these 3023 dogs were processed using the same bioinformatics pipeline referenced above. Variants private to the affected dog were prioritized by predicted consequence and impact by Variant Effect Predictor.^9^ High (eg, frame shift, loss or gain of stop or start codon, affecting a splice junction) and moderate impact (eg, missense) variants were confirmed by visual inspection using the Integrative Genomics Viewer^10^ to determine if they were, in fact, real and private variants or the result of sequencing artifacts, poor coverage over the location, or difficulty with automatic calling of structural variants. Visual confirmation assessments were evaluated by using the variant filtering steps shown in Table 1 and recorded within Table S1.

**Table 1:** Variant filtering steps.

** Filtering step**	**Heterozygous variants**	**Homozygous variants**
**Private variants**	2 68 799	78 215
**Protein-changing private variants**	507	134
**Duplicated variants (multiple transcripts) removed**	236	59
**chrUn (chromosome unknown) variants removed**	125	14
**Visually confirmed variants (IGV)**	20	4
**Missense private variants**	17	3
**High-impact protein-changing private variants**	3	1

A list of the private coding variants at each step is provided in Table S1. The resulting list of 24 variants contained 22 characterized genes. The variants (19 missense, 3 high impact) were submitted to VarElect^11^ for gene prioritization (Table S2) Missense variants were evaluated for pathogenicity with the programs MutPred2,^12^ SNPs&GO,^13^ and Provean^14^ (scores recorded within Table S1).

**Figure 1:**
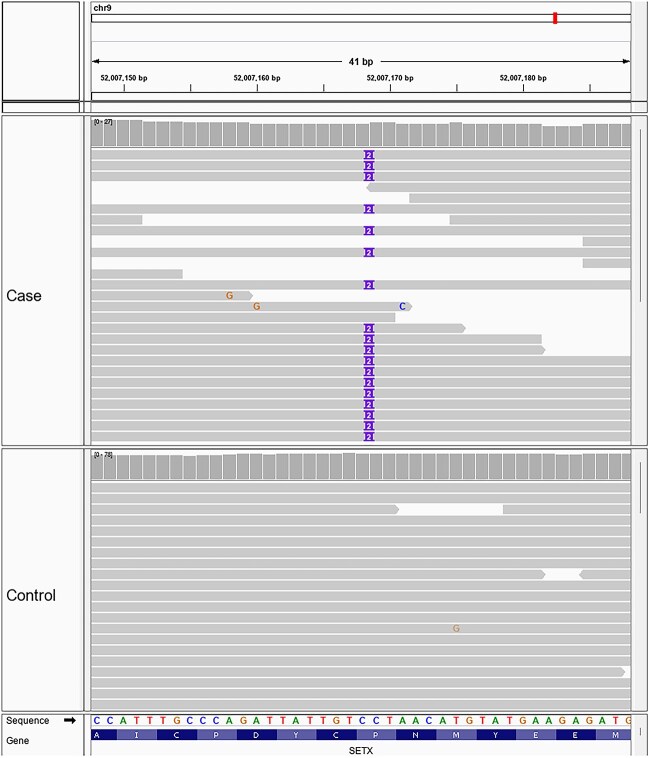
A private homozygous 2 bp insertion (UU_Cfam_GSD_1.0/canFam4-chr9:52,007,168C > CCT, XP_038404865.1, p.Asn388LeufsTer9) was identified in the *SETX* gene leading to a frameshift/premature stop codon variant that is predicted to truncate about 85% of the senataxin protein using in silico programs.

**Figure 2:**
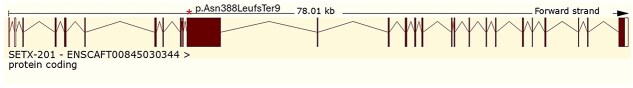
Schematic graph of the variant location. ^*^ Red star marks the location of the variant. The first changed amino acid is at position 388 (1 codon after the 2 bp insertion occurs) Asn > Leu and is predicted to terminate after 9 amino acids.

Four high-impact variants were identified within *SETX*, *LOC102156976*, *OR6C75*, and *NEDD4*, respectively. A private homozygous 2 bp insertion at UU_Cfam_GSD_1.0/canFam4-chr9:52,007,168C > CCT, *SETX* p.Asn388LeufsTer9 (XP_038404865.1) was identified leading to a frameshift/premature stop codon variant that is predicted to truncate about 85% of the senataxin protein using in silico programs (Figure 1). A schematic graphic of the location of the variant^15^ is shown in Figure 2. This variant was not found in the University of Minnesota internal WGS database described above, and the *SETX* gene had the highest VarElect ranking with submitted phenotypes including gait abnormality, hyperflexion, muscle atrophy, and weak patellar reflexes. The other 3 high-impact genes were eliminated because *LOC102156976* is an uncharacterized gene; *OR6C75* encodes an olfactory receptor commonly mutated in dogs; and *NEDD4* was deemed less likely to be the causal variant due to its much lower VarElect score.

**Figure 3:**
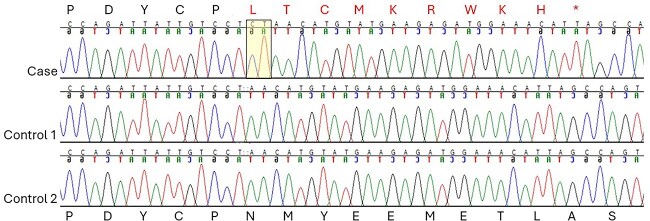
SETX Sanger sequence chromatograms of the clinical case and 2 controls showing the amino acid changes and premature stop codon. The 2 bp insertion is highlighted in yellow.

Since there were no other JRTs in our reference population and to verify that the *SETX* variant wasn’t a common allele within the breed, EDTA whole blood was submitted from 12 Belgian JRTs with various neurological and medical disorders (Table S3). The blood samples were left over from clinical cases, and IACUC approval and owner consent for use of the remaining blood were not required. DNA was purified using Qiagen Puregene (Qiagen, Germantown, MD) according to package instructions. Primer3Plus (https://www.primer3plus.com/) was used for primer design.^16^ Genotyping was performed using Sanger sequencing of a 460 bp amplicon. This PCR utilized standard conditions with forward primer 5′-AGCAAATGGAAGGAAGACCA-3′ and reverse primer 5′-GGCATCTGTTTGGTTGAGGA-3′. Sanger sequence chromatograms of the affected dog and controls are shown in Figure 3. The *SETX* 2 bp insertion was not identified in any of the 12 unrelated Belgian JRTs; all 12 of these dogs were homozygous wild type.

## Discussion

In this report, we confirm the first homozygous frameshift/premature stop codon variant in *SETX* that is the likely cause of chronic and progressive gait abnormalities in an adult JRT. The variant identified was not found in a heterozygous or homozygous state in any of the dogs of various breeds in the internal WGS database or in the group of genotyped Belgian JRTs, although the number of JRTs genotyped was not large. Based on these studies, we conclude that the *SETX* variant is likely causative in our affected JRT.

Senataxin is an RNA:DNA helicase that is important in the resolution of RNA:DNA hybrids referred to as R-loops and is relevant to DNA repair.^17^^,^^18^ Loss-of-function *SETX* variants are associated with high R-loop levels resulting in neurodegenerative diseases such as motor neuron degeneration and cerebellar neuron degeneration. Accumulation of R loops results in genomic instability due to DNA double-strand breaks, hyper-variation, hyper-recombination, transcript-associated recombination, gross chromosomal rearrangements, and chromosome loss.^19^^,^^20^

Although SETX protein expression is known to be ubiquitously distributed across tissues, variants in the *SETX* gene specifically affect neurons and are associated with different neurodegenerative diseases in humans such as gain of function variants resulting in autosomal dominant amyotrophic lateral sclerosis 4 (ALS4) and autosomal dominant spinal muscular atrophy (SMA), and loss-of-function variants resulting in autosomal recessive ataxia with oculomotor apraxia type 2 (AOA2).^21^^,^^22^ The variant identified in this case would produce a loss of function like that of AOA2 in people. In our case, the loss of function occurs at amino acid 388 out of 2699, leading to a predicted truncation of > 85% of the protein. The specific loss-of-function *SETX* variant reported in our case has not yet been identified in human patients, although several loss-of-function *SETX* variants have been described.^21^

In one report, neurological and biochemical characteristics of 13 human patients with *SETX* genetic variants were described.^21^ All presented with gait abnormalities during the second decade of life (age range from 11 to 18 years). Mild choreic movements and strabismus were noted in 2 cases before the onset of gait abnormalities. Eight of the 13 patients were wheelchair-bound after a disease duration ranging from 7 to 28 years. Mild elevation in CK activity was found in 5 patients and serum alfa-fetoprotein was elevated in all cases. In all cases, EMG showed axonal sensorimotor polyneuropathy predominantly in the lower limbs; however, spontaneous electrical activity typical of denervation was found in only 5 of 10 patients tested. When brain MRI was performed, moderate to marked cerebellar atrophy was most prominent in the vermis and mild to moderate brainstem atrophy was noted in 5 cases. Of interest, 3 additional patients in this study without a genetic diagnosis had a late onset in the fifth to seventh decade of life with a slowly progressive cerebellar syndrome and mild to markedly increased serum α-fetoprotein concentration.

In our case, gait abnormalities were not reported until approximately 7 years of age and the presence of mild or subclinical abnormalities at an earlier age cannot be ruled out. Although other systemic abnormalities were not reported, it is not clear if they were investigated. Many neurodegenerative diseases have an older age of onset such as Huntington’s disease, Parkinson’s disease, and adult-onset motor neuron diseases. In loss-of-function SETX variants with R-loop accumulations, the time to onset could be variable and the buildup of the R-loops might be progressive. It is theoretically possible that it takes time, perhaps on the order of years, for the accumulation of unstable R loops and many metabolic processes to come into play, which could explain the older age-of-onset of this dog’s clinical signs. Electromyography did not show any evidence of myokymia or myotonia and was electrically silent. This contrasts with myokymia and neuromyotonia described in the JRT breed where myokymic discharges were common; however, several JRTs were identified where the EMG was silent.^4^ Electromyographic changes were mostly seen when the dogs were showing myokymia and neuromyotonia, or neuromyotonia, during the clinical examination, sedation or rarely anesthesia. This distinction supports the importance of electrodiagnostic testing in the evaluation of movement disorders or gait abnormalities in the JRT breed and in dogs in general. In the human study of SETX patients,^21^ EMG changes typical of denervation were not found in half the patients even though the clinical phenotype was similar to the other SETX patients.

No specific abnormalities were identified in muscle biopsies using a standard panel of histochemical stains and reactions including fiber typing in cryosections of muscle biopsy samples. This tends to be a common finding among canine movement disorders and helps to distinguish this group of myopathies from other degenerative myopathies where pathological changes are common. Histopathology of muscle biopsies was not reported in the human cases.^21^

Despite the likely importance of the identified variant, there are limitations to this study. The owners had no information on the parents or littermates of this dog; thus, the genotype and clinical status of relatives are not known. A brain MRI was not performed; thus, cerebellar or brainstem atrophy was not evaluated. In addition, the dog was lost to follow-up, and a necropsy examination of the brain, spinal cord, and peripheral nerves was not performed. Concentrations of α-fetoprotein are elevated in all SETX human cases.^21^ Identification of a new *GRID2* genetic variant with progressive ataxia^22^ and a review of α-fetoprotein in progressive ataxias^23^ revealed elevated α-fetoprotein. These findings could provide the impetus to evaluate α-fetoprotein as a marker in dogs with progressive ataxia and gait abnormalities. In our JRT case, α-fetoprotein was not measured. The identification of this *SETX* variant should, however, expand our knowledge of gene variants in canine gait abnormalities.

## Supplementary Material

N13886_May_5th_2010_video_1_def_aalag085

N13886_May_3rd_2011_video_2a_walk_def_aalag085

N13886_May_3rd_2011_video_2b_trot_def_aalag085

March_8th_2013_video_3_def_aalag085

Supplemental_Table_S1_Revised_JRT_Private_Coding_Variants_aalag085

Supplemental_Table_S2_VarElect_Results_aalag085

Supplemental_Table_S3_JRTs_Belgium_aalag085

## Off-label antimicrobial declaration

The authors declare no off-label use of antimicrobials.

## Institutional animal care and use committee or other approval declaration

The authors declare no institutional animal care and use committee or other approval was needed.

## Abbreviations

bp: base pair
CK: creatine kinase
EMG: electromyography
JRT: Jack Russell Terrier
WGS: whole genome sequence
